# The productivity impact of short-term labor mobility across industries

**DOI:** 10.1007/s11187-022-00610-z

**Published:** 2022-04-02

**Authors:** Mariacristina Piva, Massimiliano Tani, Marco Vivarelli

**Affiliations:** 1 Department of Economic Policy, Università Cattolica del Sacro Cuore, Piacenza, Italy; 2 UNSW Canberra, Canberra, Australia; 3 IZA, Bonn, Germany; 4 UNU-MERIT, Maastricht, The Netherlands; 5 Department of Economic Policy, Università Cattolica del Sacro Cuore, Milano, Italy

**Keywords:** Business visits, Intrapreneurship, Labor mobility, Knowledge transfer, Productivity, O33, J61

## Abstract

The restrictions on labor mobility imposed in the COVID-19 pandemic heighten the need to review in detail the role of mobility in improving productivity and fostering economic growth. In this study, we carry out a comprehensive analysis of business visits (BVs) understood as a productivity-enhancing intrapreneurial strategy, using the most extensive set of data available, covering 33 sectors and 14 countries during the period 1998–2013. Our database merges unique information on expenditures on BVs by sector, country, and year, sourced from the US National Business Travel Association, with OECD and World Bank productivity data. We find that BVs raise labor productivity in a significant way, but short-term labor mobility exhibits decreasing returns, being more crucial in those firms, sectors, and countries characterized by less mobility and by lower productivity performances.

## Abstract (plain language summary)

COVID-19 has imposed restrictions on labor mobility and this turns out to be relevant for productivity and economic growth. Business visits (BVs) are a proper proxy (unique information is taken from the US National Business Travel Association) to measure short-term mobility. Sectoral data in 14 countries over the period 1998–2013 are used. Results do reveal that BVs have a positive impact on labor productivity. However, this effect is more relevant in those sectors and economies at a lower stage of productivity evolution, i.e., the lower the initial productivity level, the larger the effect of BVs on productivity. Therefore, BVs play a relevant role in sectors and countries lagging behind in terms of productivity. Policy makers should foster, in the post-pandemic recovery, short-term labor mobility through adequate incentives and tax exemptions, particularly in those sectors where BVs are less frequent and where productivity growth is below the average.

## Introduction

Restricting peoples’ mobility has been imposed as an effective policy measure to safeguard the public against the COVID-19 virus in several countries; however, shutting down short-term labor mobility across space^1^ (from now on: business visits, BVs) heightens the need to review in detail the role of this kind of mobility in improving productivity and fostering economic growth.

Indeed, over the decades preceding the eruption of the pandemic, intrapreneurship (Burgelman, 1983; Ireland et al., 2009; Pinchot, 1985), has benefited by a rapidly expanding range of innovation sources. In particular, companies have progressively moved away from the post-WWII model of centralized R&D activity to take advantage of globalization as an effective channel to tap knowledge, resources, and expertise located around the world (Baldwin & von Hippel, 2011; Criscuolo, 2005). For instance, firms and nations have benefited from collaborations involving exchanges of experts (Edler et al., 2011), and externalities from returning entrepreneurs (Filatotchev et al., 2011), researchers (Jonkers & Cruz-Castro, 2013; Kogut & Macpherson, 2011), and scientists (Gibson & McKenzie, 2014).

However, while long-term mobility of knowledge workers has been recently recognized as an important intrapreneurial strategy (Braunerhjelm et al., 2018), short-term mobility (BVs) has received less attention. Yet, BVs may be an important channel of knowledge transfer, being technological transfer and the exchange of tacit knowledge (Polanyi, 1966) some of the pillars of intrapreneurship (Antoncic & Hisrich, 2001; Parker, 2011). Moreover, since SMEs are generally characterized by a systematic underinvestment in fostering internal knowledge and in-house R&D (see Acs & Audretsch, 1990; Cohen & Klepper, 1996; Conte & Vivarelli, 2014; Molodchik et al., 2021), this type of knowledge transfer turns out to be particularly crucial for this category of firms.

Indeed, the (scarce) extant literature (see next section) has shed some light on the important role played by BVs as a channel through which knowledge is exchanged and created, with substantive positive effects on patenting (Hovhannisyan & Keller, 2015, 2019), productivity (Dowrick & Tani, 2011; Piva et al., 2018), and economic growth (Andersen & Dalgaard, 2011). Moreover, it was also observed that when mobility was restricted, for example through visa restrictions (Lawson & Roychoudhury, 2016; Neumayer, 2010), growth and innovation were negatively affected (Arif, 2019; Orazbayev, 2017), mainly because fewer face-to-face interactions curbed opportunities to form and exchange tacit knowledge (Katz & Martin, 1996; Scellato et al., 2015; Stephan, 2010).

Yet, despite their positive economic effects, BVs remain an overlooked topic in innovation and intrapreneurial studies. The main reason behind the status quo is likely associated with the general lack of data on the phenomenon: Short-term labor movements are not captured by the Community Innovation Surveys (CIS), and aggregate data cannot be disentangled in national and international statistics about people’s flows. In addition, at the firm level, mobility-related expenditures are merged with other administrative and general expenses. In such context, without precise information, it is challenging to understand whether BVs are mere consumption, which simply raises the utility of the individuals practicing them (Anderson et al., 2006), or rather an intrapreneurial investment to reach and absorb innovation-enhancing knowledge. This uncertainty was never examined in detail by the literature and in practice: Managerial and budget decisions about mobility continued to be dispersed across several local functional and administrative areas rather than being taken “holistically” with the intrapreneurial viewpoint of the entire organization in mind, contributing to possible duplications, inaction, and wastage (Welch et al., 2007).

In this framework, our paper aims to present and discuss the results of an extensive analysis of the productivity effects of short-term labor mobility (relative to alternative and well-known channels such as R&D expenditures), using comprehensive data and merging unique commercial information on expenditures in BVs by sector and country for 15 years during 1998–2013—sourced from the US National Business Traveller Association (NBTA)—with OECD and World Bank data on productivity, R&D expenditures, and international trade. The resulting panel database covers 33 sectors in manufacturing and services in 14 countries, in Europe and the USA.

Our study makes a number of contributions. First, it identifies short-term labor mobility using unique data on expenditures on BVs. This information, produced and distributed by NBTA (2010) to commercial airliners to forecast demand for travel, is constructed bottom-up from actual expenditures at sector-country level combined with input–output data from national accounts. Although the data are not immune from measurement error, they provide much improved identification of short-term mobility relative to the proxies used by the literature, which include the volume of tourist flows or work-related visits as recorded on landing cards. An additional advantage of using expenditures is their measurement in units that are directly comparable with well-known inputs of innovation, such as R&D investments, easing comparisons and interpretations.

Second, it uses perhaps the most extensive longitudinal set of productivity and input data covering more than 3500 sector-country-year data points, enabling one to apply comprehensive statistical techniques. Existing studies are instead constrained by data trade-offs, using in-depth sectoral or cross-country information but rarely offering both.

Third, it investigates the direct effects of short-term mobility in promoting productivity across different industries as well as the possibility of decreasing returns both in terms of business visit intensity and in terms of productivity performance. This is important to design policy for nations and strategic decisions for firms, as decreasing returns make short-term mobility strategically more relevant for disadvantaged countries and firms as SMEs. In this respect, we single out a new possible driver of SMEs’ high-growth patterns, additional to the ones identified by the extant literature on the subject (such as innovation and early internationalization, see Hölzl & Janger, 2013; Audretsch, et al., 2014; Dalgıç & Fazlıoğlu, 2021; Mogos et al., 2021).

We find that BVs positively and significantly affect productivity growth across different industries. However, they exhibit decreasing returns, being more crucial in those sectors characterized by less mobility and by lower productivity performances, such as in the early stages of the competition process or in industries mainly populated by SMEs, characterized by limited endowments of human capital and resources.

The rest of the paper is organized as follows: Section 2 reviews the literature. Section 3 discusses the empirical strategy. Section 4 presents the data. Section 5 illustrates the results. Section 6 concludes and discusses some managerial and policy implications.

## Literature review

Mobility is not a new phenomenon,^2^ though its spread has historically been constrained by technology and other factors, such as transport costs and institutional barriers. Positive technological shocks leading to better and cheaper transportation and communication have typically lifted the number of people using mobility to take up economic opportunities around the globe without having to permanently migrate. For example, the advent of steamships in the nineteenth century encouraged seasonal migration, as agricultural workers became able to be employed throughout the year following different harvest seasons in the North and South hemispheres (Piore, 1979). The arrival of commercial jet flights, in 1959, made long-distance short-term mobility easier, enabling firms to enter new markets by temporarily deploying key personnel without having to reproduce structure, functions, and positions of the head company in each location (Moss-Kanter, 1995; Ohmae, 1990; Rogers, 1995; Salt, 1992). Short-term mobility nowadays is shared by a large portion of the labor force, especially if highly educated, contributing to “grease the wheels” of global supply chains to serve customers all over the world (Roper et al., 2008; Roy et al., 2004).

Short-term mobility however has not only changed the way in which goods and services are produced and delivered: Key has been and remains its influence on how these are developed. Interacting through short-term mobility establishes opportunities of knowledge exchange between individuals within and across firms, enabling them, crucially, to form new links between what one already knows and what one learns as a result of the interaction and the steps leading to it. These novel linkages expand problem-solving capabilities and skills within individuals and organizations, raising at once the efficient absorption of new information (Cohen & Levinthal, 1989; Crescenzi & Gagliardi, 2018; Rodrigo-Alarcón et al., 2020; Teece et al., 1997), and the stimulation of creativity (Shalley et al., 2004) and learning capabilities (McCombs, 1991). Recognizing useful external knowledge and exploiting it can give firms an edge over their competitors, and new products have been shown to incorporate knowledge that exists or was originally produced outside the successful innovator (March & Simon, 1958; Mueller, 1962; Mansfield, 1968; Rosenberg & Steinmuller, 1988).

As knowledge is not uniformly distributed in space, mobility solves the strategic need to access it, by either co-locating in certain places (Bathelt et al., 2004; Florida, 2002; Howells, 2002; Torre & Rallet, 2005; von Hippel, 1987) or interacting, often face-to-face, with the individuals holding valuable embodied knowledge (Bathelt & Schuldt, 2008; Boschma, 2005; Dahl & Pedersen, 2004; Franco & Filson, 2000; Hamermesh, 2006; Polanyi, 1966; Singh, 2005; Zellner, 2003).

Despite its theoretical and anecdotal importance, empirical research on short-term mobility is dampened by the lack of data. Innovation surveys, the primary source of innovation statistics, do not include short-term mobility as a possible category among the sources contributing to product or process innovations (OECD, 2005). Short-term movements are merged within the definition of “international visits”^3^ followed by the United Nations (United Nations, 1998), but these figures are highly aggregated^4^ and do not include the main purpose for travel. Data from passenger surveys and tourism statistics are also too aggregate to inform beyond major airport destinations, average length of stay, and expenditure (e.g., IATA, 2007; ONS, 2001; ABS, various years). They also do not provide any information about the industry of the traveler. Primary data collected through in-depth interviews are highly informative and support the hypothesis that mobility is mostly carried out to exchange knowledge (Tani, 2014), but their results are typically based on too few observations to be generalized or merged with official data on productivity at sectoral or firm level. Even financial statistics from public and private database, such as Dun & Bradstreet,^5^ do not disentangle expenditures for short-term mobility from other general expenses.

Facing these constraints, the empirical literature has used proxies of short-term mobility, such as tourist (Andersen & Dalgaard, 2011; Hovhannisyan & Keller, 2015) or migration flows (Dowrick & Tani, 2011; Rogers, 1995) as well as primary data (Moss-Kanter, 1995; Salt, 1992; Tani, 2014), but each of these carries significant limitations. Tourists travel for pleasure rather than work, and visit locations hosting attractions of historical significance, natural beauty, and recreation rather than business headquarters, production facilities, or university campuses. Migration flows do not necessarily reflect business visits within or across supply chains. Primary data reflect circumstances specific to the time and location of the data collection, raising doubts about their representativeness of more general flows of BVs.

Three articles provide fundamental background to our study, as they focus on the impact of BVs on productivity. The first, Andersen and Dalgaard (2011), used travel data for 72 countries over 2 years (120 observations in total) sourced from the World Tourism Organization (UNWTO) to link international arrivals plus departures to total factor productivity (TFP) and showed that travel intensity accounts for almost 50% of the variation in aggregate TFP (OLS estimates). The possible endogeneity of travel intensity is addressed by using predicted travel shares as instruments; in particular, the 2SLS estimates imply that an increase of 10% in the travel share leads to a 0.2% increase in the level of TFP.

The second, Dowrick and Tani (2011), used cross-sectoral data within one country (Australia) measuring the specific number of business visits, as reported by arrival and departure cards over the period 1991–2005 (143 observations). In their short-term panel estimations, they found that a 10% rise in the gross flows of BVs in an industry increases multifactor productivity in that industry by about 0.1%. They also find that the productivity effect of outgoing BV is about double those of incoming BVs (0.2% vs. 0.1%).

A common main limitation of both the previous studies is the small number of observations used in their panels, which obviously constrains the power of the statistical tests performed and the reliability of the results obtained.

The third study, by ourselves (Piva et al., 2018), used unbalanced panel data on 16 sectors in 10 countries during the period 1998–2011, suggesting that mobility through BVs was indeed an effective mechanism to improve labor productivity: The estimated elasticity (0.053) is about half as large as investing in R&D, which researchers and policy makers alike generally see as the prime mechanism to foster productivity.

The present study builds on Piva et al. (2018) with the following substantial extensions. First, dealing with an extended longitudinal dataset, we have increased the number of available observations from 2049 to 3574. Second, in this study we control for the role of capital formation, R&D expenditures, and also for the possible role of trade as a channel of knowledge diffusion. Third, we dig into the investigation of the nature of the productivity impact of BVs, looking for the possibility of decreasing returns both in terms of business visit intensity and in terms of productivity performance (see next sections).

## Empirical specification

We apply and test a simple model of knowledge transfer, based on Hall and Mairesse (1995), which considers industry *i* of country *j*, which at time *t* produces value added *Y*_*ijt*_ according to the production function (1):

1 $$Y_{ijt}=AC_{ijt}^\alpha L_{ijt}^\beta\left({\textstyle\sum_r}K_{rijt}^{\gamma_r}\right)e^{b_{ij0}+\lambda_it+\varepsilon_{ijt}}$$

where *C*_*ijt*_ and *L*_*ijt*_ are the industry input of physical capital and labor, respectively, and ${K}_{rijt}^{{\gamma }_{r}}$ represents the level of productive knowledge available to the industry via activity *r*: *K*_*rijt*_ includes knowledge-enhancing activities like R&D expenditures, spending on short-term labor movements, and international trade in goods and services; the parameter ${\gamma }_{r}$ represents the proportional increase in productive knowledge resulting from the *r*th activity (*r* = 1, 2, …). Finally, the last factor captures other productivity drivers, including an initial industry-specific and country-specific level of value added *b*_*ij0*_, a deterministic time trend *λ*_*i*_*t* representing the exogenous growth of the global technological frontier in a given industry (*λ*_*i*_ being the rate of disembodied technical change), and an idiosyncratic error term *ε*_*ijt*_.

Transforming (1) in logarithmic form and rearranging it to measure value added per employee yield the following (2):

2 $${y}_{ijt}-{l}_{ijt}=a+\alpha \left({c}_{ijt}-{l}_{ijt}\right)+{\gamma }_{1}\left({k}_{1ijt}-{l}_{ijt}\right)+{\gamma }_{2}\left({k}_{2ijt}-{l}_{ijt}\right)+\dots +\left(\alpha +\beta +{\gamma }_{1}+{\gamma }_{2}+\dots {\gamma }_{t-1}-1\right){l}_{ijt}+{\gamma }_{r}{k}_{rijt}+\dots +{b}_{0ij}+{\lambda }_{i}t+{\varepsilon }_{ijt}$$

where *y*, *l*, *a*, *c*, and *k*_*r*_ represent the natural logarithms of *Y*, *L*, *A*, *C*, and *K*_*r*_.

Empirically, we focus on the estimates of the parameters ${\sum }_{r}{\gamma }_{r}$ to assess the role of the alternative channels affecting labor productivity (value added per employee); this means to estimate the following testable specification (3):

3 $${\mathrm{ln}\left(\frac{VA}{E}\right)}_{ijt}= \mathrm{constant}+\alpha {\mathrm{ln}\left(\frac{C}{E}\right)}_{ijt}+{\gamma }_{1}{\mathrm{ln}\left(\frac{K}{E}\right)}_{ijt}+{\gamma }_{2}ln{\left(\frac{BV}{E}\right)}_{ijt} {+(\alpha +\beta +{\gamma }_{1}+{\gamma }_{2}-1)\mathrm{ln}(E)}_{ijt}+ {\gamma }_{3}{\mathrm{ln}\left(\frac{X+M}{\mathrm{GDP}}\right)}_{ijt}+{b}_{{0}_{ij}}+{\lambda }_{i}t+{\varepsilon }_{ijt}$$

with *i* (sector) = 1,…, 33; *j* (country) = 1,…, 14; *t* (time) = 1998,…, 2013; ln = natural logarithm.

Productivity is measured by labor productivity (value added, VA, over total employment, E), while our control impact variables are the physical capital stock (C) per employee, the R&D stock (K, for knowledge) per employee, and the trade intensity (import plus export over GDP^6^). The measure of our key impact variable is the whole business visit (BV) stock per employee.

Taking per capita values permits both standardization of our data and elimination of possible sector/country size effects. In this framework, total employment (E) is a kind of control variable: In case (*α* + *β* + γ_1_ + γ_2_ − 1) turns out to be greater than zero, it indicates increasing returns in the labor input.

As it is common in this type of literature (Hall et al., 2009; Heshmati & Kim, 2011; Kumbhakar et al., 2012; Mohnen & Hall, 2013; Ortega-Argilés et al., 2010, 2014, 2015; for a different approach using a dynamic specification in flows, see Damioli et al., 2021), stock indicators rather than flows should be considered impact variables; indeed, productivity is affected by the accumulated stocks of different inputs and not only by volatile current or lagged flows. Furthermore, dealing with stocks rather than flows has two additional advantages: First, since stocks incorporate the accumulated investments in the past, the risk of endogeneity is minimized; second, there is no need to deal with the complex and arbitrary choice of the appropriate lag structure for the flows.

The stocks are computed following the perpetual inventory method (PIM):

4 $${S}_{t0}=\frac{{INV}_{t0}}{\left(g+\delta \right)}; {S}_{t1}={S}_{t0}\left(1-\delta \right)+{INV}_{t1}$$

where *S* is the stock, *INV* measures the investment flow, *δ* is a depreciation rate (6% for capital stock; 15% for knowledge capital stock; 15% for business visits stock^7^), and *g* is computed as an “ex post” 3-year compound growth rate.

## Data

We use a unique commercial database developed by the US National Business Travel Association to forecast trends in international short-term mobility after 9/11. Following that event, travel to the USA reduced considerably and NBTA members (most air carriers around the world) were especially worried about the future demand for travel. As a result, the NBTA embarked on a major, and to date unique, exercise to gather detailed information on travel expenditures by industry and country to develop a new database to forecast future travel expenditures. This database was compiled using statistics on travel services recorded in each country’s national input–output tables and sources such as various Ministries for Tourism, airlines ticket sales, and IATA (International Air Transport Association).

We combine this unique database with public OECD and World Bank data on productivity, R&D expenditures, and international trade for the period 1998–2013.

An important advantage of using travel expenditures rather than people’s flows is the possibility to compute the elasticity of a dollar spent on BVs on productivity, which can be compared with the corresponding estimates of elasticity for other knowledge production activities such as R&D expenditures.

In more detail, data on business travel expenditures are annual information available for 48 sectors of 72 countries over the period 1998–2013. The data aggregates expenditures made by incoming and outgoing domestic and international travelers in a given industry-country-year cell, and is reported in current US$.

As far as the other variables are concerned, value added, physical investments, R&D expenditures, and employment are taken from OECD sources. In particular, OECD-STAN is the source for most of the information, merged with OECD-ANBERD as far as R&D is considered. Harmonized OECD STAN and ANBERD sectoral data, based on the two-digit ISIC Rev. 4 industrial classification, are available over the 1998–2013 time span for the following countries: Austria, Belgium, the Czech Republic, Finland, France, Germany, Hungary, Italy, Norway, Portugal, Slovakia, Sweden, the UK, and the USA. The final panel, merging data from NBTA and OECD, is unbalanced (due to OECD missing values) and covers a total of 3574 longitudinal observations. All the monetary series have been corrected for purchasing power parities, expressing, at the end, values in constant prices and PPP 2010 US dollars. Moreover, in order to control for an additional channel of technology transfer, we also considered a trade variable at the country level (measured as (Export + Import)/GDP).^8^

The sample composition by countries and by sectors is presented in Tables 1 and 2.

**Table 1:** Sample composition by countries

Country	Observations
Austria	304
Belgium	230
Czech Republic	479
Finland	280
France	186
Germany	369
Hungary	428
Italy	310
Norway	320
Portugal	259
Slovakia	63
Sweden	140
UK	20
USA	186
**Total**	**3574**

**Table 2:** Sample composition by sectors

Industries	ISIC Rev. 4	Observations
Agriculture, forestry, and fishing	01–03	149
Mining and quarrying	05–09	47
Food products, beverages, and tobacco products	10–12	149
Textiles	13	70
Wearing apparel	14	75
Leather and related products, footwear	15	62
Wood and products of wood and cork, except furniture; articles of straw and plaiting materials	16	139
Paper and paper products	17	67
Printing and reproduction of recorded media	18	113
Coke and refined petroleum products	19	32
Chemicals and chemical products and basic pharmaceutical products and pharmaceutical preparations	20–21	145
Rubber and plastic products	22	93
Other non-metallic mineral products	23	106
Basic metals	24	89
Fabricated metal products, except machinery, and equipment	25	181
Computer, electronic and optical products	26	183
Electrical equipment	27	183
Machinery and equipment n.e.c	28	183
Motor vehicles, trailers, and semi-trailers	29	87
Other transport equipment	30	182
Furniture; other manufacturing; repair and installation of machinery and equipment	31–33	158
Electricity, gas, and water supply; sewerage, waste management, and remediation activities	35–39	55
Construction	41–43	190
Wholesale and retail trade, repair of motor vehicles and motorcycles	45–47	161
Transportation and storage	49–53	62
Accommodation and food service activities	55–56	105
Telecommunications	61	110
IT and other information services	62–63	86
Financial and insurance activities	64–66	97
Real estate activities	68	18
Scientific research and development	72	165
Public administration and defense; compulsory social security	84	16
Education	85	16
**Total**		**3574**

Some descriptive statistics and preliminary univariate correlation coefficients are reported in Table 3.

**Table 3:** Descriptive statistics and correlation matrix

	Mean (St. deviation)	ln(VA/E)	ln(C/E)	ln(K/E)	ln(Trade)	ln(BV/E)
ln(VA/E)	4.22 (0.58)					
ln(C/E)	4.06 (1.89)	0.527*				
ln(K/E)	1.94 (1.61)	0.613*	0.501*			
ln(Trade)	4.39 (0.44)	− 0.200*	− 0.415*	− 0.185*		
ln(BV/E)	1.83 (1.38)	0.542*	0.565*	0.393*	− 0.320*	
ln(E)	4.52 (1.53)	− 0.116*	0.032	− 0.307*	− 0.434*	− 0.167*

Employees are expressed in thousands of persons engaged, and monetary variables are expressed in millions of constant PPP 2010 US dollars. Trade is the sum of exports and imports of goods and services measured as a share of GDP

^*^ Significant at 95%

## Results

Specification (3) has been estimated through different econometric methodologies. Firstly, pooled ordinary least squared (POLS) regressions have been run to provide preliminary evidence. Even if simple, POLS regressions have been controlled for two sets of dummies (country and time, turning out to be always jointly significant, as shown in Table 4,^9^) and for heteroscedasticity (robust standard errors).

**Table 4:** Dependent variable: ln (value added per employee)

	(1) POLS	(2) FE	(3) POLS	(4) FE
ln(C/E)	0.452*** (0.011)	0.131*** (0.023)	0.421*** (0.012)	0.078*** (0.023)
ln(K/E)	0.037*** (0.005)	0.098*** (0.012)	0.046*** (0.006)	0.101*** (0.012)
ln(Trade)	0.158* (0.081)	0.227*** (0.036)	0.132* (0.081)	0.141*** (0.035)
ln(BV/E)			0.006*** (0.001)	0.021*** (0.002)
ln(E)	0.033*** (0.006)	0.014 (0.024)	0.048*** (0.006)	0.169*** (0.026)
Constant	1.212*** (0.083)	3.457*** (0.177)	1.285*** (0.082)	2.578*** (0.186)
Time dummies	Yes	Yes	Yes	Yes
Country dummies	Yes	-	Yes	-
Time-dummies Wald test (*p*-value)	2.30*** (0.003)	6.64*** (0.000)	2.63*** (0.000)	10.21*** (0.000)
Country-dummies Wald test (*p*-value)	165.66*** (0.000)	-	129.24*** (0.000)	-
Hausman test (*p*-value)		10.35*** (0.000)		43.90*** (0.000)
Adj. *R*^2^ *R*^2^ within	0.73	0.27	0.74	0.30
Number of country/sector units of observation	287			
Number observations	3574			

* Significant at 90%; ** significant at 95%; *** significant at 99%

Secondly, fixed effect (FE) regressions have been performed in order to take into account sector-specific unobservable time-invariant characteristics. When different sectors are not pooled together, estimates control for unobserved heterogeneity as well as for within-sector path dependence (see Capone et al., 2019). The shortcoming is that constant variables—such as country belonging—are no longer individually identified, as they are encompassed by the individual sector-level fixed effects.

Thirdly, random effect (RE) regressions have also been ran and tested versus the FE specification. According to the outcomes of the Hausman test (see Table 4), the FE estimates are preferable to the RE ones.

Columns 1 and 2 in Table 4 report the results from the estimates without the BV stock. These are in line with the previous literature about the link between physical capital and R&D on the one side and productivity on the other side: Physical capital appears to have a positive and highly significant impact on productivity with an elasticity ranging from 0.131 to 0.452; at the same time also knowledge capital shows a positive and highly significant impact ranging from 0.037 to 0.098.^10^ As expected, our trade control participates to increase labor productivity with a highly significant coefficient in the preferred FE estimation.

When the BV stock per employee is added to the estimated specification, previous results are substantially confirmed (columns (3) and (4)).

Focusing on our key variable, the impact of the BV stock per employee on productivity turns out to be positive and statistically significant at the 99% level of confidence in both the estimates, ranging from 0.006 to 0.021 (POLS vs. FE). This outcome supports the view that productivity is also significantly explained by the expenditures devoted to the business visits, although this additional impact is lower in magnitude than those originated by capital formation, knowledge capital, and trade: If a given company doubles its BV stock per employee, productivity is expected to grow by about 2%. Overall, these results are also consistent with the literature presented in Section 2.

To better evaluate the presence of possible different impacts of BVs, we study the possibility of decreasing returns both in terms of business visit intensity and in terms of productivity performance. Hence, we split the sample into two subsamples on the basis of the average business visit intensity (BV/E) at the sectoral level. Choosing this strategy allowed us having two comparable subsamples including 17 industries for the low BV-intensive aggregate and 16 industries for the high one.^11^

Estimates have been run using the preferred FE specification. Results presented in Table 5 are, in general, consistent with the previous ones (with the exception of the loss of statistical significance of the physical capital in the low BV-intensive industries). Focusing on the magnitude of the BV effect, it turns out an impact of 0.027 in the low-intensive industries and an impact of 0.011 in the high-intensive ones (both highly significant). These results tend to support a decreasing returns interpretation: The lower the starting level of business visits per capita, the higher their impact on productivity. This is consistent with the powerful effect of the initial face-to-face contacts and interactions, bringing a significant impact especially at the starting stage of the investment in short-term mobility.

**Table 5:** Dependent variable: ln (value added per employee)

	(1) FE Av.BV/E < 10.000	(2) FE Av.BV/E > = 10.000
ln(C/E)	− 0.033 (0.030)	0.162*** (0.034)
ln(K/E)	0.135*** (0.016)	0.080*** (0.017)
ln(Trade)	0.186*** (0.043)	0.109** (0.057)
ln(BV/E)^§^	0.027*** (0.002)	0.011*** (0.002)
ln(E)	0.076** (0.035)	0.229*** (0.039)
Constant	3.354*** (0.252)	2.266*** (0.271)
Time dummies	Yes	Yes
Time-dummies Wald test (*p*-value)	6.23*** (0.000)	4.63*** (0.000)
*R*^2^ within	0.44	0.22
Number of sectors	17	16
Number observations	1847	1727

^*^Significant at 90%; ** significant at 95%; *** significant at 99%

^**§**^The test on the significance of the difference of ln(BV/E) coefficients in (1) and (2) is equal to 52.02***, meaning that the null of no significant difference is strongly rejected

As a robustness check, we run a regression where both groups (below and above the average business visit intensity) are included in a single equation with an indicator for being more or less BV-intensive.^12^ Results are displayed in Table 7 in the Appendix and are fully consistent with those reported in Table 5. Finally, in order to be more granular in considering the different ranges of BV intensity, we also run—in a similar fashion to what reported in Table 7—a regression including five interaction variables based on the distribution of the BV intensity along its five quintiles. Results are presented in Table 8 and—consistently with our interpretation in terms of decreasing returns—the magnitude of the relevant coefficient turns out to be monotonically decreasing moving from the first to the fifth quintile.

As a further extension of the analysis, we analyze the relationship between BVs and productivity using a quantile regression based on the productivity level. As the panel is unbalanced, we applied a quantile estimator controlling for country and time dummies.^13^ Results turn out to be generally consistent with the ones in Table 4. Nevertheless, additional evidence has emerged as BVs tend to have a positive impact on productivity, but with an almost monotonically decreasing effect moving from the first quantile to the last one (see Table 6). This suggests an effect of BVs more important for productivity laggers than for productivity champions. While this outcome cannot be interpreted as a further evidence of decreasing returns *stricto *sensu, it appears consistent with a context where BVs exert their more powerful productivity impact in weaker economic contexts, such as industries characterized by a large presence of SMEs.

**Table 6:** Dependent variable: ln (Value Added per employee)

	(1) Quantile 0.2	(2) Quantile 0.4	(3) Quantile 0.6	(4) Quantile 0.8
ln(C/E)	0.282*** (0.007)	0.325*** (0.007)	0.436*** (0.010)	0.497*** (0.009)
ln(K/E)	0.099*** (0.004)	0.086*** (0.004)	0.042*** (0.005)	0.010** (0.005)
ln(Trade)	0.156** (0.075)	0.258*** (0.064)	0.199*** (0.063)	0.180*** (0.069)
ln(BV/E)^§^	0.008*** (0.001)	0.005*** (0.001)	0.003*** (0.001)	0.003*** (0.001)
ln(E)	0.093*** (0.005)	0.073*** (0.005)	0.054*** (0.004)	0.035*** (0.005)
Constant	2.157*** (0.137)	2.551*** (0.116)	2.419*** (0.110)	2.414*** (0.123)
Time dummies	Yes	Yes	Yes	Yes
Country dummies	Yes	Yes	Yes	Yes
Time-dummies Wald test (*p*-value)	6.77*** (0.000)	4.88*** (0.000)	6.43*** (0.000)	4.06*** (0.000)
Country-dummies Wald test (*p*-value)	120.09*** (0.000)	194.83*** (0.000)	181.64*** (0.000)	264.89*** (0.000)
Pseudo *R*^2^	0.52	0.51	0.51	0.52

^*^Significant at 90%; ** significant at 95%; *** significant at 99%

^§^Tests were run comparing adjacent coefficients among quantiles. Results show they are always significantly different (at 99%). The results are available from the authors upon request

## Conclusions and managerial and policy implications

This paper reviews existing evidence and offers new, comprehensive results supporting that BVs increase productivity and enhance technology transfer. Together with capital formation, R&D expenditures, and trade, BVs play a substantive role in positively and significantly affecting productivity growth across different industries and countries.

This outcome is consistent with the extant industry-based empirical studies as well as the scarce microeconomic literature on the subject. In addition, we find robust and novel evidence that BVs exhibit decreasing returns, being more crucial in those sectors characterized by less mobility and by lower productivity performances, such as in sectors and firms disadvantaged by size, location, or limited endowments of social and human capital (this is the standard situation which may occur in SMEs, see Sterlacchini, 1999; Santarelli & Tran, 2013).

Our general result reflects that BVs remain fundamental to generate knowledge exchanges even when alternative forms of communication exist (e.g., videoconferencing): BVs enable face-to-face interactions and these make participants decide immediately whether to trust each other (Gambetta, 1988; Storper & Venables, 2004). If mutual trust is established, then reciprocal understanding and cooperation can increase, as the transaction costs and uncertainty associated with sharing knowledge are lower, facilitating exchanges of information (Amin & Cohendet, 2004; Hansen, 1999) and the creation of “social capital” and networks (Burt, 1997; Dosi et al., 2020; Portes, 1998). Starting new interactions by distance mode is unlikely to be ever as effective, though they may be the only viable alternative when short-term labor mobility is halts because of lockdowns or other restrictions to movement.

Therefore, the outcomes of this study strongly support the hypothesis that mobility offers firms and nations disadvantaged by geography, size, or historical circumstances a way to access the talent and knowledge necessary to kick-start or catch-up (Lee, 2019; Porto et al., 2021) and uplift productivity and economic conditions. As short-term mobility is relatively simple to implement vis-à-vis productivity channels that require large initial costs or expertise (e.g., R&D), it has the advantage of being a flexible and effective tool to access or share “sticky” productive knowledge.

In turn, these considerations carry interesting managerial and policy implications.

Within organizations, general managers might make a conscious strategic use of the productivity-enhancing role of BVs. Short-term mobility should be thought as an intrapreneurial investment at the company’s level and not just as a localized choice undertaken by decision nodes scattered across the organization. Moreover, our results indicate that those firms less investing in BVs and less productive are likely to be the ones that can benefit more from engaging in this particular type of investment. This paves the way to a specific policy focus on SMEs and less productive companies, in order to foster their opening in terms of incoming and outgoing BVs. In this respect, the outcomes of this study reveal that an intrapreneurial strategy based on fostering BVs is even more crucial for SMEs rather than for large corporations that are generally thought as the natural environment where intrapreneurial activities can take place.

At the national level, our results suggest that short-term labor movements are not only consumption expenditures that can be taxed at will, but also an intrapreneurial investment in knowledge-enhancing activities. In this respect, mobility could be gainfully embraced to foster human capital growth, as in the case of Europe’s Erasmus program (Ackers, 2005), as well as technology transfer and productivity gains. Therefore, policy makers should foster short-term labor movements through adequate incentives and tax exemptions, particularly in those sectors where BVs are less frequent and where productivity growth is below the average.

Furthermore, unlike migration and long-term assignments and relocations, short-term mobility amplifies a nation’s endowment of human capital without permanently affecting its people’s headcount. Knowledge exchanges arising from in-bound and out-bound movements cannot be netted out in some unlikely mobility-related knowledge balance, and as such they offer both origin and destination firms and countries the opportunity to enhance their productivity; in other words, mobility is far from being a zero-sum game where there are net importers and exporters of knowledge.

## Data Availability

NBTA data is proprietary, and OECD and World Bank data are publicly available.

## Appendix

**Table 7:** Dependent variable: ln (value added per employee)

ln(C/E)	0.105*** (0.012)
ln(K/E)	0.072*** (0.023)
ln(Trade)	0.148*** (0.035)
ln(BV/E) low-intensive (17 industries)	0.026*** (0.001)
ln(BV/E) high-intensive (16 industries)	0.013*** (0.002)
ln(E)	0.158** (0.026)
Constant	2.710*** (0.185)
Time dummies	Yes
Time-dummies Wald test (*p*-value)	10.38*** (0.000)
*R*^2^ within	0.32
Number observations	3574

* Significant at 90%; ** significant at 95%; *** significant at 99%

**Table 8:** Dependent variable: ln (value added per employee)

ln(C/E)	0.112*** (0.012)
ln(K/E)	0.065*** (0.023)
ln(Trade)	0.109*** (0.036)
ln(BV/E) first quintile	0.026*** (0.002)
ln(BV/E) second quintile	0.020*** (0.001)
ln(BV/E) third quintile	0.019*** (0.001)
ln(BV/E) fourth quintile	0.019*** (0.001)
ln(BV/E) fifth quintile	0.015*** (0.001)
ln(E)	0.133*** (0.026)
Constant	2.812*** (0.186)
Time dummies	Yes
Time-dummies Wald test (*p*-value)	9.99*** (0.000)
*R*^2^ within	0.32
Number observations	3574

* Significant at 90%; ** significant at 95%; *** significant at 99%

## Footnotes

1 BVs are a form of labor’s geographic mobility rather than mobility across jobs.

2 Throughout history, people living in settled societies have been moving around the globe for economic reasons. At times these movements consisted of relocating from one area to another, typically after environmental, health, and man-made events that disrupted economic activity, such as droughts, plagues, and wars and persecutions. More generally they involved returning to the place of origin where consumption or revenue generation would ultimately materialize. Examples of this form of movement, which is referred to as mobility, include traveling in search of, or to supply, tradable commodities or services, information, and employment. There is evidence of Greek merchants from the cities of Attica trading with remote regions in the third century BC, of managers of the Medici’s bank traveling across cities in Northern Europe to keep in touch with business conditions in the fourteenth century, and of Marco Polo’s travels to and then throughout China to inform the Khan about business environment within his empire (http://www.let.leidenuniv.nl/history/migration/index.html).

3 An *international visitor* is defined by the UN as “any person who travels to a country other than that in which he/she has his/her usual residence but outside his/her usual environment for a period not exceeding 12 months and whose main purpose of visit is other than the exercise of an activity remunerated from within the country visited” (UN, 1998—para. 29). The category of international visitors includes tourists (overnight visitors) and same-day visitors (also known as “excursionists”) (UN, 1998—para. 30).

4 Data on movements at international level reflects an anachronistic convention to classify movements between two countries according to the length of stay (UN, 1998): Movements can be either “visits” if they involve a change in the “usual residence” for less than 12 months and no payment is received from the host country, or “migrations” if they last for over 1 year. Migration in turn is divided into “long-term” if there is a change of usual residence longer than 12 months and “short-term” when the change of residence lasts between 3 and 12 months. “Temporary migration” is also used at times to define particular categories of stays that grant employment rights and last a number of years (typically up to four), depending on the host country’s regulations. These nevertheless are reclassified as visits or migrations in international statistics depending on their length of stay. This classification tends to be followed by national statistical offices, although this is not always the case making it challenging to obtain consistent historical series (e.g., Salt et al., 1994; OECD, 2008). As some visits allow recipients to subsequently apply for permanent visa, since 2006 the OECD (SOPEMI reports) has reclassified a number of visits into permanent movements if the underlying entry visa had either no expiry date, could be renewed indefinitely, or allowed recipients to apply for permanent residence in the host country.

5 Dun & Bradstreet is a US-based private corporation that offers credit and financial information (including accounts), on more than 300 million businesses around the world: https://www.dnb.com/.

6 Since we do not adjust this macroeconomic indicator by employee but rather by national GDP, we do not add ${\gamma }_{3}$ to the coefficient $()$. This is the reason why we isolated *k*_*rijt*_ in Eq. (2).

7 This is what is assumed by the reference literature, taking into account that the knowledge capital (in our case both R&D expenditures and business visits) exhibits a faster degree of obsolescence rather than the physical capital (see Nadiri & Prucha, 1996 for singling out 6% as the proper discount rate for physical capital; Hall, 2007 and Hall et al., 2009 for proposing 15% as the standard discount rate for R&D).

8 This macroeconomic control turns out to be the same for all the sectors within a given country in a given year.

9 Country and time dummies control for other determinants of productivity growth such as the initial level of value added and the advances in the global technological frontier (see Eq. (1)).

10 These magnitudes are quite consistent with the extant literature, reporting estimated elasticities of productivity to R&D ranging from 0.05 to 0.25.

11 The 17 industries in the low BV-intensive aggregate are as follows: agriculture, forestry, and fishing; mining and quarrying; wearing apparel; leather and related products, footwear; chemicals and chemical products and basic pharmaceutical products and pharmaceutical preparations; basic metals; fabricated metal products, except machinery and equipment; machinery and equipment n.e.c.; motor vehicles, trailers, and semi-trailers; furniture; other manufacturing; repair and installation of machinery and equipment; construction; wholesale and retail trade, repair of motor vehicles and motorcycles; accommodation and food service activities; IT and other information services; financial and insurance activities; public administration and defense; compulsory social security; and education. The 16 industries in the high BV-intensive aggregate are as follows: food products, beverages, and tobacco products; textiles; wood and products of wood and cork, except furniture; articles of straw and plaiting materials; paper and paper products; printing and reproduction of recorded media; coke and refined petroleum products; rubber and plastic products; other non-metallic mineral products; computer, electronic and optical products; electrical equipment; other transport equipment; electricity, gas, and water supply; sewerage, waste management, and remediation activities; transportation and storage; telecommunications; real estate activities; and scientific research and development.

12 Practically, two interaction dummies allow the two groups to have different coefficients within a common specification using all the 3574 available observations.

13 To give a broad idea of the country representativeness in the quantiles (although industries of the same country might belong to different quantiles), in the quantile 0.2—in terms of overall observations and considering the most represented three countries—Hungary accounts for 33%, the Czech Republic 31%, Portugal 19%; in the quantile 0.4, Germany 18%, the Czech Republic 16%, Italy 13%; in the quantile 0.6 Finland 14%, Norway 14%, Austria 13%; in the quantile 0.8 Belgium 12%, Norway 12%, the USA 12%.

## Code availability

Stata codes.
